# A Promising Future for Prostate Cancer Diagnostics

**DOI:** 10.3390/diagnostics7010006

**Published:** 2017-01-17

**Authors:** Stephen J. Assinder, Vanitha Bhoopalan

**Affiliations:** Discipline of Physiology, School of Biomedical Sciences and Bosch Institute, University of Sydney, Sydney, NSW 2006, Australia; vbho4415@uni.sydney.edu.au

**Keywords:** PI-RADS, diffusion weighted imaging, ^223^RaCl_2_, [^18^F]-NaF PET/CT, PERCIST, circulating tumour cells, oncosomes, oncosomal DNAs and RNAs, quantum dot multiplex arrays, carbon nanotube technologies

## Abstract

It has been estimated that globally there is a death attributable to prostate cancer every four minutes. As life expectancy in all world regions increases, so too incidence of this disease of the ageing male will increase. For many men diagnosis occurs after presentation with symptoms of altered urinary dynamics. Unfortunately, these changes, whilst also associated with benign disease, are evident quite late in the aetiology of prostate cancer. Early detection provides for better management and prognosis. This Special Issue provides an up to date view of the advances made towards early diagnosis and prognosis. It provides reviews of advanced imaging techniques (e.g., multiparametric MRI and protocols), and of biomaterials and molecular biomarkers currently being explored (e.g., microRNAs, proteomics) and the technologies that are revolutionizing this field. It describes the multi-disciplinary approaches that are essential to inexpensive, deliverable and accurate platforms for prostate cancer diagnostics.

## 1. Introduction

World Health Organisation (WHO) statistics demonstrate that global life expectancy of males at birth has increased from 65.8 years to 69.1 years between 2005 and 2015. Indeed, life expectancy has increased in all world regions (range 2.2–6.5 years; WHO statistics, 6 June 2016 http://apps.who.int/gho/data/view.main.SDG2016LEXREGv?lang=en). As prostate cancer (PCa) is a cancer of the ageing male, when considered in the context of statistics of all cancers globally, there is no doubt that it will become a major contributor to the approximate “doubling by 2030 of annual cancer cases” as predicted from data of the International Agency for Research on Cancers [1]. Indeed, consistent with this prediction are recent figures from the Global Burden of Disease Collaboration that show a 33% increase in cancer cases between 2005 and 2015 with population ageing being the greatest contributing factor [2]. It is interesting to note that the greatest increase in male life expectancy has occurred in the African region (6.5 years). Similarly, in the period between 2007 [3] and 2012 [4] prostate cancer has become the most common cancer of men in the African region. For developing countries prostate cancer was reported to have risen from the 6th most common cancer in 2007 [3] to being the 4th most common cancer in 2012 [4]. Age standardized rates (disability-adjusted-life-years) of prostate cancer are greatest in countries of low sociodemographic index [2]. Hence, as the number of diagnoses will undoubtedly increase then the need for more refined approaches to better discriminate disease and inform the treatment strategy most suited to the individual becomes paramount.

Towards addressing this need, two emerging areas in diagnosis and prognosis are discussed in this Special Issue. Advances in imaging technologies are presented in the review by Bourne and Pananagiotaki and in two studies provided by Kairemo and colleagues. Advances in potential molecular biomarkers of prostate cancer are presented by Vlaeminck Guillem, who provides a comprehensive overview of a range biomarkers that can be assayed from blood, whilst Tonry and colleagues provide a review of technologies that assess multiple protein markers of PCa towards individualized prostate cancer care.

## 2. Imaging Local and Bone Metastasized Prostate Cancers

Problems with sensitivity of traditional diagnostic methods have long been recognized to have major implications of overtreatment and associated issues of unnecessary morbidity and costs. Magnetic resonance imaging (MRI) has been shown to have a promising future in diagnosing clinically significant prostate cancers. To provide a standardized approach to multi parametric magnetic resonance imaging (mp-MRI) of prostate cancer the Prostate Diagnostic Imaging Consensus Meeting (PREDICT) panel recommended a Likert-scale reporting system [5]. Two retrospective studies demonstrated a positive correlation with tumour volume and Gleason score [6,7], where a Likert score of 3 or more is the strongest predictor of targeted biopsy positivity and clinically significant PCa [7]. Similarly, in 2012 the European Society of Urogenital Radiology (ESUR) published guidelines for the use of mp-MRI in the detection and characterization of prostate cancer lesions [8] with the introduction of the Prostate Imaging Reporting and Data System (PI-RADS), a scoring system for mp-MRI which has recently been updated (PI-RADSv2) [9]. A retrospective analysis has shown that a PI-RADSv2 score of 4 or greater could preoperatively predict clinically significant PCa [10]). A recent prospective study, however, provides evidence that PI-RADSv2 criteria “result in a high-false positive rate” therefore decreasing the detection rate of significant PCa where scores are below 5 [11].

Whilst these and several other recent studies (e.g., Lee and colleagues [12], and Radtke and colleagues [13]) have gone some way towards standardizing prostate mp-MRI interpretation improvements can still be made. Since the publication of the ESUR guidelines a number of studies have illustrated that, of the three components of mp-MRI, diffusion weighted imaging (DWI) is by far the best predictor of the size and grade of prostate cancer lesions. The article by Bourne and Panagiotaki (this issue) discusses the evidence for this and provides a concise and elegant description of DWI and how it works to detect changes in tissue structure during tumorigenesis. The authors highlight that DWI is sensitive to “arrangement, type, geometry and permeability of cells at the micron scale”, a utility that is often not appreciated. Despite these significant advantages, the article also provides the reader with a balanced critique of the limitations of current recommended DWI protocols. It presents the assumptions underlying the apparent diffusion coefficient model that leads to a lack of biological specificity and discusses phenomenological models, and the predictive value and increased diagnostic accuracy that is gained by combining parameters of each model.

Whilst mp-MRI is focused on detection and staging of PCa, combined positron-emission tomography/computed tomography (PET/CT) and gamma imaging provide approaches that can exploit radiotracers that target anatomical, pathological and physiological features of PCa to evaluate distant metastases. Kaimero and colleagues present two studies with respect to these imaging techniques. Metastatic castrate resistant prostate cancers (mCRPC) present a significant challenge to disease management. Bone metastases are common in mCRPC patients and the primary cause of morbidity and mortality of this group. The recent introduction of the bone seeking radium-223-dichloride radionuclide has heralded a new era in the management of mCRPC patients. ^223^RaCl_2_ is an α-emitting analogue of calcium that is incorporated into areas where there is increased bone turnover through hydroxyapatite complexing. It was approved by the FDA in 2013 due to its ability to significantly increase overall survival, but also provides additional benefits such as bone pain reduction. The dosing regimen of this drug is based on the findings of the ALSYMPIA trial (cited by Kairemo et al., this issue). Kairemo and colleagues respond to recent European legislation which requires that pre-treatment dose-planning is undertaken in the use of radiopharmaceuticals by providing a method that uses γ-scintigraphy monitoring of ^223^RaCl_2_ labeling of bone lesions to calculate relative dose and monitor therapy. Two case studies are presented that suggest utility in determining response or progression. In one case a new lesion was detected in the absence of clinical symptoms during the treatment course. Diagnostic imaging with PET/CT has gained popularity in PCa diagnostics with the radiotracers [^18^F]-fluorocholine and [^18^F]-NaF [14]. [^18^F]-fluorocholine targets prostate cancer cells to image lesions in the prostate gland, nodal invasion and distant metastases. [^18^F]-NaF labels sites of bone metastases due to its incorporation into fluorapatite (the same way it is in the process of tooth remineralisation as catalysed by fluoride in fluoridated water or toothpastes) during bone remodeling. Kairemero and Joensu (this issue) provide a retrospective analysis of patients who had undergone ^223^RaCl_2_ therapy and who had been monitored by [^18^F]-NaF PET/CT. The authors demonstrate the detection of a significant treatment response by [^18^F]-NaF PET/CT analysis alone, and which is supported by S-PSA dynamics. Importantly, this is the first time that this approach has been applied to evaluation of ^223^RaCl_2_ therapy response. The authors note the conservative nature of PET Response Criteria in Solid Tumours (PERCIST) stating that, of all those patients determined as responding to ^223^RaCl_2_ therapy by [^18^F]-NaF PET/CT, only half of the patient cohort were determined as responding favourably according to PERCIST. It is suggested that the approach presented provides a simple, routine procedure for analyzing skeletal malignant activity in response to common combination therapies. The reader is referred to Wahl et al. [15] for review and comparison of Response Evaluation Criteria in Solid Tumours (RECIST) and PERCIST.

## 3. Molecular Diagnostics

Molecular markers also have utility for detection of secondary tumours and tumour recurrence. Their greatest promise, however, is in the development and provision of screens to predict risk of development of significant PCa. They are amenable to inexpensive platforms that can be provided by primary healthcare professionals easily and that do not require expensive imaging methodologies restricted to urban conurbations less readily accessed by persons in rural or remote regions. In this Special Issue, two comprehensive reviews are provided by Vlaeminck-Guillem, who describes a number of circulating biomarkers that can be assayed from blood, and by Tonry and colleagues, who present an updated discussion of proteomics in the diagnosis and prognosis of prostate cancer. Both provide a timely update to prior reviews such as that of Velonas et al. [16].

Vlaemick-Guillem (this issue) provides an overview of the variety of materials “shed” by tumours that are a rich well-spring for biomarker development. The review describes some of the recent advances in the accurate methods now available for detection of PCa derived circulating materials. For example, the only clinically validated FDA approved test for circulating tumour cells (CTC) is the CellSearch (Veridex) system. Appropriately, the author highlights some of the compromises made by such systems, such as sensitivity and purity, and that CTC based methods are poor indictors of early stage PCas. Whilst the use of CTC methodologies are unlikely to satisfy screening parameters, they might provide valuable tools for monitoring disease progression and treatment response. As well as CTCs other circulating materials of tumour cell origin provide a rich source of “molecular cargo”. These circulating microvesicles released from PCa cells are tumour specific and can be isolated from biological fluids. Those exosomes released from prostate epithelium were first described in seminal fluid and shown to provide biological functions to the ejaculate in reproductive physiology [17]. It is now recognized that PCas shed large extracellular vesicles termed “large oncosomes” that contain proteins, recycled lipids and a range of nucleotides that present a unique tumour cell signature that can be exploited in the development of diagnostic biomarkers [18]. Vlaenick-Guillem provides a review of those cell-free, oncosomal DNAs and RNAs (including microRNAs) that potentially provide diagnostic and prognostic potential. Of those circulating miRNAs listed by Vlaemick-Guillem, miR-21, miR-141 and miR-221 are most frequently reported as deregulated with circulating miR-141 consistently increased in men with high risk PCa, metastatic PCa and castrate resistant PCa. It is important to note that these miRNAs have also been shown to be altered in tissue biopsies [19,20,21] and, at least for miR-21, in urine [19]. When combined with two other miRNAs enriched in urine (miR-19a and miR-19b) miR-21 has greater diagnostic power than PSA [19]. Such studies have highlighted the value of less invasive biological fluids sources of biomarkers than blood or tissue biopsies for the diagnosis and prognosis of prostate cancer.

In their comprehensive review of technologies that assess multiple protein markers Tonry and colleagues (this issue) discuss the less invasive biological fluid sources of semen and urine based biomarker assays. The authors describe the well-known combined protein:genetic based PCA3-*TMPRSS2:ERG* validated urine test that can stratify PCa without the need for biopsy as well as the more recent SelectMDX mRNA based assay, amongst others. Proteomics and proteins are likely to provide the most convenient biomarkers for assay of appropriate biological fluids (blood, urine, semen) on easy to use platforms in a primary healthcare situation. In their description of biomarker discovery, Tonry and colleagues provide an up-to-date review of proteomic technologies that provide utility in this regard. Of particular promise are those technologies that are nanotechnology based and aptamer based. Nanotechnologies are particularly exciting as they have the potential to provide highly sensitive, miniaturized, low-cost and primary healthcare solutions for PCa Diagnostics. The review describes a number of developments in this field including the use of quantum dot multiplex arrays and carbon nanotube technologies (CTNs). Indeed, CTNs have been used to measure the matrix metalloproteinase 2 (MMP2) in serum. As MMP2 is commonly elevated in metastatic PCas, this at least provides in-principal proof.

## 4. Conclusions

This Special Issue of prostate cancer diagnostics highlights two important aspects: (1) the approaches to prostate cancer diagnostics are developing rapidly; and (2) these approaches are multi-facetted and multidisciplinary. There is little doubt that the progress being made is proceeding rapidly due to the multidisciplinary approaches being taken. Furthermore, a multidisciplinary approach is most likely to succeed in providing an inexpensive, deliverable and accurate screen for PCa that best informs treatment choices and subsequent methods of treatment monitoring. On the basis of the contributions in this Special Issue, the future of prostate cancer diagnostics is very promising.
